# Assessment of research productivity of Arab countries in the field of infectious diseases using Web of Science database

**DOI:** 10.1186/2049-9957-4-2

**Published:** 2015-02-02

**Authors:** Waleed M Sweileh, Samah W Al-Jabi, Alaeddin Abuzanat, Ansam F Sawalha, Adham S AbuTaha, Mustafa A Ghanim, Sa’ed H Zyoud

**Affiliations:** Department of Pharmacology and Toxicology, College of Medicine and Health Sciences, An-Najah National University, Nablus, Palestine; Department of Clinical and Community Pharmacy, College of Medicine and Health Sciences, An-Najah National University, Nablus, Palestine; Department of Microbiology and Immunology, College of Medicine and Health Sciences, An-Najah National University, Nablus, Palestine; Department of Biochemistry and Genetics, College of Medicine and Health Sciences, An-Najah National University, Nablus, Palestine

**Keywords:** Bibliometric, Infectious diseases, Arab world, Web of science

## Abstract

**Background:**

To meet the future challenges of infectious diseases and limit the spread of multidrug resistant microorganisms, a better understanding of published studies in the field of infectious diseases is needed. The objective of this study was to analyze the quantity and quality of research activity in the field of infectious diseases in Arab countries and compare it with that in non-Arab countries.

**Methods:**

Documents published in Arab countries within the research category of “infectious diseases” were extracted and analyzed using the Web of Science database. The data analyzed represent research productivity during the time interval between 1900 – 2012.

**Results:**

Worldwide, the total number of documents published in the field of infectious diseases up to 2012 was 227,188. A total of 2,408 documents in the field of infectious diseases were published in Arab countries, which represents 1.06% of worldwide research output. Research output from Arab countries in the field of infectious diseases was low for decades. However, approximately a five-fold increase was observed in the past decade. Arab countries ranked 56^th^ to 218^th^ on the standard competition ranking (SCR) in worldwide publications in the field of infectious diseases. Egypt, with a total publication of 464 (19.27%) documents ranked first among Arab countries, while Kuwait University was the most productive institution with a total of 158 (6.56%) documents. Average citation per document published in Arab countries was 13.25 and the *h*-index was 64. Tuberculosis (230; 9.55%), malaria (223; 9.26%), and hepatitis (189; 7.8%) were the top three infectious diseases studied as according to the retrieved documents.

**Conclusion:**

The present data reveals that some Arab countries contribute significantly to the field of infectious diseases. However, Arab countries need to work harder to bridge the gap in this field. Compared with non-Arab countries in the Middle East, research output from Arab countries was high, but more efforts are needed to enhance the quality of this output. Future research in the field should be encouraged and correctly directed.

**Electronic supplementary material:**

The online version of this article (doi:10.1186/2049-9957-4-2) contains supplementary material, which is available to authorized users.

## Multilingual abstracts

Please see Additional file 1 for translation of the abstract into the six official working languages of the United Nations.

## Background

According to the Arab Union League, there are 22 independent Arab states with more than 400 million inhabitants. Poverty, inadequate health services, political instability, and outbreaks of infectious diseases are common in many Arab countries [1–3]. For example, there was an outbreak of polio in Syria that made international agencies call for a ceasefire to accomplish a polio vaccination campaign [4]. Another example is the cholera outbreak in Baghdad after the second Gulf War [5]. In Saudi Arabia, several mortalities have been attributed to Middle East respiratory syndrome caused by the coronavirus infection [6–8]. Furthermore, pilgrimage and other religious seasons create a true challenge for Saudi Arabia to prevent and control outbreaks of infectious diseases during religious seasons [9–11]. Different types of infectious diseases are prevalent and have become a health burden for governments in many Arab countries. For example, according to the World Health Organization (WHO), in conservative communities that Arab countries fall into, HIV infections are rising at a faster rate, while the coverage of antiretroviral therapy is the lowest [12]. Furthermore, highly pathogenic and serious viral infections such as the avian flu virus, hepatitis B, and hepatitis C are important risks of morbidity and mortality, and pose real threats in some Arab countries such as Egypt [13–17]. Similarly, serious parasitic infections such as malaria, schistosomiasis, and trypanosomiasis constitute a major health, social, and economic challenge in Egypt, Sudan, Yemen, and other Arab countries [18–23]. Zoonotic infections, such as brucellosis and hydatid disease, are also present in several Arab countries and pose a continuous health challenge [24–28]. Furthermore, some infectious diseases such as nematode infections, filarial infections, schistosomiasis, fascioliasis, leprosy, and trachoma are endemic in some Arab countries and are being neglected [29]. In addition to the abovementioned challenges pertaining to infectious diseases, there is evidence that serious and common infectious agents in the Arab region, such as *Mycobacterium tuberculosis, Staphylococcus aureus*, and some gram-negative bacilli are developing multiple drug resistance which is a real future public health challenge at regional and global levels [30–38].

To meet future challenges regarding infectious diseases and limit the spread of multidrug resistant microorganisms in Arab countries, a better understanding of published studies in the field of infectious diseases is needed to have baseline data in this field so that future research can be encouraged and correctly directed. No bibliometric studies have addressed microbiological, or parasitological or viral or infectious diseases research activity in the Arab region. Most bibliometric studies in Arab countries have focused on biomedical research in general or on other medical areas [39, 40]. Therefore, this study was carried out to investigate the quantity and quality of research activity in Arab countries in the field of infectious diseases. This bibliometric study assessed the past and current research activity in Arab countries in the field of infectious diseases, in order to draw future attention to research in this field.

## Methods

The Web of Science (WoS) database was used to achieve the objective of this study. The WoS is a trustworthy, large, and powerful database for literature retrieval and analysis [41]. All Arab countries: Kingdom of Saudi Arabia (KSA), Egypt, Jordan, Lebanon, Qatar, Bahrain, Kuwait, Morocco, Tunisia, Syrian Arab Republic (SAR), United Arab Emirates (UAE), Iraq, Sudan, Yemen, Algeria, Comoros, Djibouti, Libya, Mauritania, Oman, Somalia, and Palestine, were used as the country keys, followed by “infectious diseases,” which was used as the WoS category. Because WoS does not recognize Palestine as an independent state yet, search for documents about infectious diseases from Palestine was carried out using separate search keys in the database.

The search keys for the 21 Arab countries looked like this: (CU = (Jordan) OR CU = (Iraq) OR CU = (Syria) OR CU = (Saudi) OR CU = (Kuwait) OR CU = (Egypt) OR CU = (Yemen) OR CU = (Qatar) OR CU = (Emirates) OR CU = (Bahrain) OR CU = (Oman) OR CU = (Sudan) OR CU = (Tunisia) OR CU = (Algeria) OR CU = (Lebanon) OR CU = (Libya) OR CU = (Morocco) OR CU = (Somalia) OR CU = (Djibouti) OR CU = (Comoros) OR CU = (Mauritania)) AND WC = (infectious diseases). The search keys for Palestine looked like this: WC = (infectious diseases) AND CI = ((Nablus) OR (Jenin) OR (Ramallah) OR (Bethlehem) OR (Tulkarm) OR (Abu Dis) OR (Gaza)) AND CU = (Israel). The search for Palestinian research output in infectious diseases was based on the list of major Palestinian cities as a key research in addition to the name of Israel as a country since the WoS does not recognize Palestine as a state and considers all Palestinian publications to be affiliated with Israel.

The results from 21 Arab countries and those from Palestine were combined and the resultant data were analyzed. To increase the accuracy of the results, research was refined and limited to original research articles and review articles because they represent actual research activities, while other types of documents such as editorials, conference proceedings, and others were excluded. The timeframe for the results included all years up to 2012. The years 2013 and 2014 were excluded in order to enhance the accuracy of the results. If we included recent years, then we might not be able to retrieve the same number of documents if we re-do the analysis several months later because some journal issues are released and uploaded to WoS several months after publication online.

The WoS generates a count of the total number of original articles, total citations, and the value of the *h*-index. Scientific output was evaluated based on a methodology developed and used in other bibliometric studies [42–47]. The collected data were used to generate the following information: (a) total and trends of contributions in infectious diseases research up until the specified date of December 31, 2012; (b) Arab countries research productivity; (c) journals in which researchers from the Arab world were published; (d) *h*-index for retrieved publications from Arab countries; and finally (e) comparison of the results obtained from Arab countries with those obtained from non-Arab countries such as Turkey, Iran, and Israel.

### Statistical analysis

Data from WoS were exported to a Microsoft Office® Excel spreadsheet and then transferred to a Microsoft Word document. Results were converted to rank order using the standard competition ranking (SCR). We took into consideration the top 10 ranked publications in each item. If the measurements of bibliometric analysis had the same ranking number, then a gap was left in the ranking numbers which followed. The journal’s impact factors (IF) were evaluated using the Journal Citation Report® (JCR; Web of Science) 2012 science edition, published by Thomson Reuters (New York, NY, USA).

## Results

The total number of worldwide documents retrieved from WoS using the methodology stated and without specifying the name of any country was 227,188. When the same methodology was applied using the list of Arab countries, 2,408 documents were retrieved. Therefore, research output from Arab countries in the field of infectious diseases represents 1.06% of worldwide research productivity. Table 1 lists the Arab countries and their standard competition rank (SCR) worldwide. In the same table, three non-Arab Middle Eastern countries are shown for comparative purposes.

**Table 1:** **List of Arab countries and three non-Arab countries in the Middle East and their Standard Competition Ranks (SCRs) in worldwide research productivity in the field of infectious diseases**

***Arab Countries***	***Worldwide SCR*** ^*^
Egypt	56
Kingdom of Saudi Arabia	60
Tunisia	68
Morocco	81
Kuwait	91
Lebanon	93
Sudan	86
Algeria	103
United Arab Emirates	105
Jordan	130
Qatar	136
Bahrain	143
Oman	157
Iraq	163
Syria	162
Yemen	175
Libya	170
Mauritania	177
Palestine	N/A*
Somalia	210
Comoros	213
Djibouti	218
**Non-Arab countries**	
Israel	25
Turkey	31
Iran	46

*Palestine is not recognized as an independent state in the WoS database, so rank could not be determine. Retrieval of documents from Palestine was explained in the methodology section.

The top 10 journals for publication of documents in the field of infectious diseases from Arab countries are shown in Table 2. Approximately 8.5% of documents from Arab countries in the field of infectious diseases were published in journals with an IF > 5. In terms of worldwide documents, approximately 21.5% were published from journals with an IF > 5.

**Table 2:** **Top 10 journals in which documents in the field of infectious diseases were mostly published from Arab countries**

***SCR*** ^***a***^	***Journal***	***Frequency N = 2408 (%)***	***IF*** ^***b***^
**1st**	*Medecine et Maladies Infectieuses*	202 (8.39)	0.753
**2nd**	*Bulletin de la Societe de Pathologie Exotique*	138 (5.73)	NA
**3nd**	*Journal of Infectious Diseases*	127 (5.27)	5.848
**4th**	*International Journal of Tuberculosis and Lung Disease*	115 (4.78)	2.610
**5th**	*Journal of Infection in Developing Countries*	98 (4.07)	0.996
**6th**	*Emerging Infectious Diseases*	80 (3.32)	5.993
**7th**	*Journal of Infection*	77 (3.20)	4.073
**8th**	*Diagnostic Microbiology and Infectious Disease*	73 (3.03)	2.260
**9th**	*Infection and Immunity*	69 (2.87)	4.074
**10th**	*International Journal of Antimicrobial agents*	67 (2.78)	4.415
**10th**	*International Journal of Infectious Diseases*	67 (2.78)	2.357

*Abbreviations*: *SCR* Standard Competition Ranking, *NA* not available, *IF* impact factor.

^a^If equal journals have the same ranking number, then a gap is left in the ranking numbers.

^b^The impact factor was reported according to the Institute for Scientific Information (ISI) journal citation reports (JCR) 2012.

**Figure 1:**
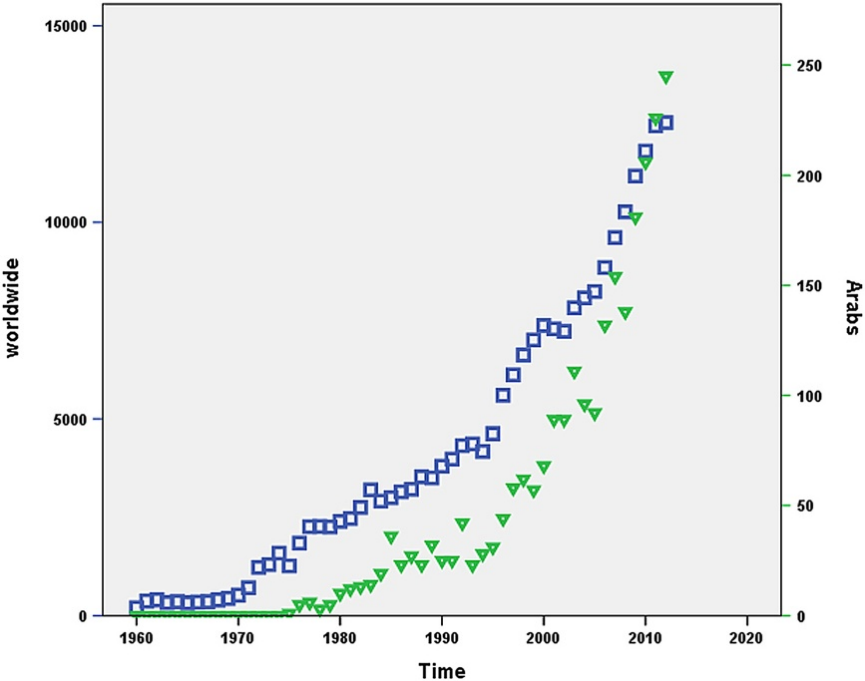
***Growth of research productivity in the field of infectious diseases.*** The □ line represents worldwide growth while the ▼ line represents growth of research in Arab countries.

The annual number of worldwide published documents was low up until 1970. A steady and obvious increase was seen after that. Growth of research in the field of infectious diseases worldwide and from Arab countries is shown in Figure 1. The figure presents the data from the 1960s to 2012. Data earlier than 1960 was excluded because the number of published documents before 1960 was relatively low. Both worldwide and Arab countries have shown a dramatic increase in research productivity in infectious diseases over time. The annual number of documents published in Arab countries indicated that research output in the field remained low until the mid-1980s and showed a great jump in the late 1990s. The first article published in the field of infectious diseases in Arab countries was co-authored by an Arab researcher from Egypt in 1928 [48]. The majority of documents retrieved from Arab countries were published in English (2,087; 86.66%), followed by French (311; 12.92%), and very few were published in Russian, Spanish, and German (10; 0.42%).

When the retrieved documents were analyzed by country, Egypt (464; 19.27%) had the highest research output, followed by the Kingdom of Saudi Arabia (437; 18.15%) and Tunisia (365; 15.16%) (see Table 3). All Arab countries, even those with low income and/or political instability such as Palestine, had some contribution to the field of infectious diseases research. The most research productive institute was Kuwait University followed by the American University in Beirut, with 158 (6.56%) and 139 (5.78%) documents, respectively. Researchers in Arab countries collaborated most with researchers from the USA (475; 19.73%), France (257; 10.67%), and England (162; 6.73%).

**Table 3:** **Research productivity of Arab and three non-Arab countries in the field of infectious diseases, including the h-index and mean citation per document**

***Arab country***	***Frequency N = 2408 (%)***	***h-index***	***Mean citation per document***
Egypt	464 (19.27)	41	16.08
Kingdom of Saudi Arabia	437 (18.15)	36	15.64
Tunisia	365 (15.16)	25	8.89
Morocco	216 (8.97)	22	9.96
Kuwait	175 (7.27)	27	17.61
Lebanon	173 (7.18)	24	14.14
Sudan	165 (6.85)	28	16.28
Algeria	134 (5.57)	16	8.06
United Arab Emirates	128 (5.32)	27	19.98
Jordan	79 (3.28)	16	12.81
Qatar	55 (2.28)	11	7.58
Bahrain	44 (1.83)	11	9.82
Oman	34 (1.41)	9	8.47
Iraq	29 (1.20)	8	11.03
Syria	29 (1.20)	10	8.21
Yemen	23 (0.96)	6	5.62
Libya	22 (0.91)	8	9.64
Mauritania	19 (0.79)	8	10.00
Palestine	11 (0.46)	4	4.91
Somalia	6 (0.25)	4	12.33
Comoros	6 (0.25)	4	5.17
Djibouti	5 (0.21)	4	8.00
***Non-Arab Countries versus 22 Arab Countries***			
**“non-Arab countries”**	2,408	64	13.25
Israel	2,095	93	24.11
Turkey	1,461	53	12.77
Iran	572	29	9.04

Of the 2,408 documents considered for the *h*-index, 64 had been cited at least 64 times at the time of the data analysis. Analysis of citation revealed that the Arab country with the highest *h*-index was Egypt (*h*-index = 41), while the country with the highest mean citation per document was the United Arab Emirates (20 citations per document). Compared with other non-Arab countries in the Middle East, the research output from Arab countries was higher than that from Israel, Turkey, and Iran. However, the quality of research output from Arab countries, measured as *h*-index, was less than that from Israel but higher than that from Turkey or Iran (see Table 3). The top infectious diseases studied as per the retrieved published documents were: tuberculosis (230; 9.55%), malaria (223; 9.26%), hepatitis (189; 7.8%), HIV (186; 7.72%), diarrhea/gastroenteritis (150; 6.23%), meningitis (115; 4.78%), leishmaniasis (111; 4.6%), salmonella (92; 3.82%), and influenza (52; 2.16%).

## Discussion

In this study, we assessed research productivity in the field of infectious diseases in Arab countries. Our results indicated that there is an obvious and promising rise in research activity in Arab countries in this field. However, none of the Arab countries ranked among the first 50 countries in terms of worldwide contribution to research output in this field. This was disappointing given that three non-Arab Middle Eastern countries—Israel, Iran, and Turkey—were ranked among the first top 50 countries. Among the Arab countries, Egypt and KSA occupied the top rank. Large populations and high national incomes are the most probable reasons for this. Compared with non-Arab countries in the Middle East, the number of published research documents in Arab countries was the highest. However, the quality of the published documents in Arab countries, as measured by the *h*-index and/or average citation per document, was lesser than that from Israel possibly because of the relatively low impact factor (IF) of journals in which the bulk of the documents from Arab countries were published. The citation is a key indicator of research quality [49]. The *h*-index was developed to overcome the main disadvantages of other bibliometric indicators, such as total number of papers or total number of citations. The *h*-index simultaneously measures the quality and quantity of scientific output, and is one of the most commonly used indicators of research quality. However, if the *h*-index is used to measure documents from different databases, then this can give different values. Therefore, each database has pros and cons when being measured by the *h*-index [50–52]. Criticisms have also been addressed to the use of the *h*-index as a marker of publication quality and citation. For example, the *h*-index does not consider the context of citations or the number of authors in the document, and gives equal values for book citation and research citations. Therefore, the *h*-index has a lesser predictive accuracy and precision than measuring using mean citations per paper, although this is controversial [53, 54].

Infectious diseases have no borders and prevention; control and eradication of infectious diseases requires worldwide efforts. Arab countries have been the source of some fatal infectious diseases, and they should heavily participate and cooperate in research in order to combat them [55–58]. For example, the Middle East respiratory syndrome was initially diagnosed in KSA but reports of cases in distant parts of the world have been identified due to people traveling from KSA [59–62]. Several research documents about serious infectious agents such as the West Nile Virus have been published in the USA and Europe through cooperation with Arab institutions and investigators [63, 64]. Arab countries should also cooperate in research regarding resistant cases of infectious agents particularly those pertaining to *Mycobacterium tuberculosis*. Emergence of resistant strains causing tuberculosis is a challenge for many developing countries [65]. Another important example of an infectious disease common in Arab countries that has been investigated through several cooperative research studies is leishmaniasis [66–68]. Cooperation in research becomes evident when we compare research interests of Arab investigators with those in other countries. Research collaboration improves the quality and quantity of research output, as well as the visibility of national health problems [69–72]. Furthermore, international collaboration in research helps in capacity building in developing countries and makes national problems of developing countries more observable [73]. Arab researchers need to take the lead and promote research projects in the field of infectious diseases as an important public health concern. Obstacles to do research such as inadequate funding, an unstable democratic atmosphere, and an unclear national policy toward health research are, unfortunately, prevalent in Arab countries. Everyone should participate in the war against infectious diseases including clerks and politicians who can help in the fight of infectious diseases such as HIV/AIDS [74, 75]. Researchers, academics, and healthcare professionals should promote vaccinations against infectious diseases as well as public hygiene as a methods of early prevention [76, 77]. Research output from a particular country is not only a point of prestige but also a reflection of the care of governments toward citizens and their individual health. Of course, the research activity and capacity of a particular country depends on several factors including the national income and the size of the population. However, in the case of infectious diseases, Arab countries, with a population of over 400 million and huge resources, must strive for excellence in research pertaining to infectious diseases. In addition, Arab countries need to invest in research in infectious diseases to limit the spread of microbial drug resistance that might be unique in different world regions. To achieve this, Arab countries must invest more in research activity in the field of infectious diseases.

Our study is the first to analyze the quantity and quality of research productivity in the field of infectious diseases in Arab countries. The database used for analysis, Web of Science (WoS), is one of the most trustworthy databases that allows for powerful citation analysis. It is well known that the most leading and influential journals in the field of medicine and health are indexed in WoS. However, articles published in non-WoS indexed journals were not included in the analysis. Despite this, the aim of this paper was to initiate discussion among professionals and academics pertaining to infectious diseases research activity in Arab countries rather than criticize or praise the research activity itself.

## Conclusion

Our study showed that research in the field of infectious diseases is rising in some Arab countries such as Egypt and KSA, and that more efforts are required to bridge the gap with some of the other countries. Research collaboration between institutions in Arab countries with international researchers and institutions in the field of infectious diseases should be sought. Governmental funding and support for infectious diseases research is also recommended.

## Electronic supplementary material

Additional file 1: **Multilingual abstracts in the six official working languages of the United Nations.** (PDF 255 KB)

## Abbreviations

WoS: Web of Science
KSA: Kingdom of Saudi Arabia
USA: United States of America
JCR: Journal citation report
SCR: Standard competition ranking
IFs: Impact factors.
