# Supplementary material for: Assessment of research productivity of Arab countries in the field of infectious diseases using Web of Science database
Source: Infect Dis Poverty. 2015 Feb 2;4(1):2. doi: 10.1186/2049-9957-4-2 (PMC4327970; doi:10.1186/2049-9957-4-2)
Supplement: Supplementary file 1 — Additional file 1: Multilingual abstracts in the six official working languages of the United Nations. (PDF 255 KB) [file 40249_2014_91_MOESM1_ESM.pdf]

Translation of the abstract into the six official working languages of the United Nations

تقييم إنتاجية البحوث في الدول العربية في مجال الأمراض المعدية باستخدام قاعدة بيانات شبكة للعلوم

وليد محمد صويلح، سماح و. الجابي، علاء الدين أبو أنسام ف. صوالحة، أدهم س. أبو طه، مصطفى أ. غانم، ح. الزيود

ملخص

خلفية: لمواجهة التحديات المستقبلية للأمراض المعدية والحد من انتشار الميكروبات المقاومة للأدوية المتعددة، هناك حاجة إلى فهم للدراسات المنشورة في مجال الأمراض المعدية بشكل أفضل. وكان الهدف من هذه الدراسة تحليل كمية ونوعية النشاط البحثي في مجال الأمراض المعدية في البلدان العربية ومقارنتها مع مثيلاتها في الدول غير العربية.

تم استخراج وثائق نشرت في الدول العربية ضمن فئة البحوث "الأمراض المعدية" وتحليلها باستخدام قاعدة بيانات شبكة للعلوم.

النتائج: في جميع أنحاء العالم، كان إجمالي عدد الوثائق المنشورة في مجال الأمراض المعدية حتى عام 2012 227188. ونشر ما مجموعه 2408 وثيقة في مجال الأمراض المعدية في الدول العربية، وهو ما يمثل 1.06٪ من مخرجات البحوث في جميع أنحاء العالم. كانت مخرجات البحوث من الدول العربية في مجال الأمراض المعدية منخفضة على مدى عقود. ومع ذلك، لوحظ زيادة قدرها ما يقرب من خمسة أضعاف في العقد الماضي.

تتراوح مرتبة الدول العربية ما بين 56 حتى 218 على مستوى المنافسة القياسي (SCR) في المنشورات العالمية في مجال الأمراض المعدية. جاءت مصر بمنشورات مجموعها 464 (19.27٪) في المرتبة الأولى بين الدول العربية، بينما كانت جامعة الكويت المؤسسة الأكثر إنتاجية مع ما مجموعه 158 (6.56٪) وثيقة. كان متوسط الاقتباس لكل وثيقة نشرت في الدول العربية 13.25وكان مؤشر هيرش 64. وتوزعت النسب على الأمراض كالتالي: السل (230؛ 9.55٪) والملاريا (223، 9.26٪)، والالتهاب الكبد الوبائي (189، 7.8٪)، حيث كانت الأمراض المعدية الثلاثة الأكثر دراسة وفقا للوثائق .

الخلاصة: تكشف البيانات الحالية أن بعض الدول العربية تساهم بشكل كبير في مجال الأمراض المعدية. ومع ذلك، تحتاج الدول العربية إلى العمل بجد من أجل سد الفجوة في هذا المجال. ومقارنة مع الدول غير العربية في الشرق الأوسط، كانت نتائج البحوث من دول عربية عالية، ولكن هناك حاجة إلى مزيد من الجهود لتحسين نوعية هذا الناتج. وينبغي تشجيع البحوث المستقبلية في هذا المجال وتوجيها بشكل صحيح.

Translated from English version into Arabic by Mahmoud Sami, through

TRANSLATORS
WITHOUT BORDERS

基于 Web of Science 数据库评价阿拉伯国家在传染病领域的科研产出

Waleed M. Sweileh, Samah W. Al-Jabi, Alaeddin Abuzanat, Ansam F. Sawalha, Adham S. AbuTaha, Mustafa A. Ghanim, Sa'ed H. Zyoud

摘要

引言：为了迎接传染病领域的挑战，限制多重耐药微生物的传播，需要进一步了解传染病领域已发表的研究资料。本研究旨在比较分析阿拉伯国家与非阿拉伯国家，在传染病领域科学研究工作数量和质量的区别。

方法：使用 Web of Science 数据库，对发表在阿拉伯国家的传染病领域的论文进行提取分析。

结果：截至 2012 年，在传染病领域，全球范围内共发表了 227 188 篇文章。阿拉伯国家共发表了 2 408 篇，占全球发文量的 1.06%。虽然阿拉伯国家在传染病领域的发文量要落后几十年，但是，在过去十年发文量大约增加了 5 倍。在传染病领域，阿拉伯国家在全球出版物标准竞争排名(SCR)为第 56 到 218 位。埃及以 464 篇（19.27%）在阿拉伯国家中名列第一，科威特大学以 158 篇（6.56%）在有论文产出的机构中名列第

一。阿拉伯国家单篇论文的平均引文率为 13.25，h 指数为 64。肺结核（230；9.55%），疟疾（223；9.26%）和肝炎（189；7.8%）是发文量位居前 3 位的传染性疾病。
结论: 目前的数据表明，一些阿拉伯国家在传染病领域贡献突出。然而，阿拉伯国家仍需更加努力缩小在这个领域的差距。阿拉伯国家的发文量高于中东的非阿拉伯国家，但仍需努力提高文章质量。应鼓励传染病领域的研究，并需正确引导。

Translated from English version into Chinese by Yi Feng-yun, through

CHINESE CENTER FOR DISEASE CONTROL AND PREVENTION
NATIONAL INSTITUTE OF PARASITIC DISEASES

Evaluation de la Productivité de la Recherche des Pays Arabes dans le Domaine des Maladies Infectieuses, en utilisant la base de données Web of Science.

Waleed M. Sweileh, Samah W. Al-Jabi, Alaeddin Abuzanat, Ansam F. Sawalha, Adham S. AbuTaha, Mustafa A. Ghanim, Sa'ed H. Zyoud

Résumé
Contexte: Afin de répondre aux défis futurs présentés par les maladies infectieuses et de limiter la prolifération de micro-organismes multi-résistants aux médicaments, une meilleure compréhension des études publiées dans le domaine des maladies infectieuses est nécessaire. L'objectif de cette étude était d'analyser la quantité et la qualité de l'activité de recherche dans le domaine des maladies infectieuses dans les pays arabes et de les comparer avec celles des pays non-arabes.
Méthodes: Des documents publiés dans les pays arabes dans la catégorie de recherche des "maladies infectieuses" ont été extraits et analysés en utilisant la base de données «Web of Science. »
Résultats: A l'échelle mondiale, le nombre total de documents publiés dans le domaine des maladies infectieuses jusqu'en 2012 a été de 227.188. Un total de 2.408 documents dans le domaine des maladies infectieuses a été publié dans les pays arabes, ce qui représente 1,06% du rendement mondial de la recherche. Le rendement de la recherche des pays arabes dans le domaine des maladies infectieuses était bas depuis des décennies. Cependant, il a été observé que ce rendement a pratiquement quintuplé au cours de la dernière décennie. Les pays arabes étaient classés de 56ème à 218ème au "Standard Competition Ranking" (SCR) dans les publications mondiales dans le domaine des maladies infectieuses. L'Egypte, avec une publication totale de 464 (19.27%) documents, était classée première parmi les pays arabes, tandis que l'Université du Koweït était l'institution la plus productive, avec un total de 158 (6.56%) documents. La moyenne des citations par document publié dans les pays arabes était de 13,25 et le *h*-index était de 64. La tuberculose (230; 9,55%), la malaria (223; 9.26%), et l'hépatite (189; 7,8%) étaient les trois maladies infectieuses principales étudiées d'après les documents récupérés.
Conclusion: Les données actuelles révèlent que certains pays arabes contribuent de manière significative dans le domaine des maladies infectieuses. Cependant, les pays arabes ont encore des efforts à fournir pour combler l'écart dans ce domaine. Comparé aux pays non-arabes du Moyen-Orient, le rendement de la recherche des pays arabes était élevé, mais davantage de travail est nécessaire pour renforcer la qualité de ce rendement. La recherche à venir dans ce domaine devra être encouragée et correctement dirigée.

Translated from English version into French by Ode Laforge, through

Оценка эффективности исследования арабских стран в области инфекционных заболеваний посредством использования базы данных Web of Science

Валид М.Свейли, Самах В.Аль-Джаби, Аладдин Абузанат, Ансам Ф.Савала, Адхам С.АбуТаха, Мустафа А.Ганим, Сайед Х.Зиуд (Waleed M. Sweileh, Samah W. Al-Jabi, Alaeddin Abuzanat, Ansam F. Sawalha, Adham S. AbuTaha, Mustafa A. Ghanim, Sa’ed H. Zyoud)

Отрывок

История вопроса: Чтобы успешно противостоять будущим вызовам инфекционных заболеваний и ограничить распространение микроорганизмов, устойчивых ко многим лекарствам, необходимо лучше понимать опубликованные исследования, касающиеся инфекционных заболеваний. Цель данного исследования состоит в изучении количества и качества научно-исследовательских работ в области инфекционных заболеваний в арабских странах, и в сравнении их с неарабскими странами.

Методы: Изданные в арабских странах документы в исследовательской категории "инфекционных заболеваний" были отобраны и изучены с использованием базы данных Web of Science.

Результаты: Общее количество документов, изданных по всему миру в области инфекционных заболеваний до 2012 года, составило 227,188. В арабских странах было опубликовано 2,408 документов в области инфекционных заболеваний, что составляет 1,06% от мирового количества исследовательских работ. На протяжении десятилетий в арабских странах издавалось небольшое количество исследовательских работ. Однако за последнее десятилетие был отмечен приблизительно пятикратный прирост. В мировых публикациях в области инфекционных заболеваний арабские страны занимали с $56^{\text{го}}$ по $218^{\text{е}}$ места по стандартной классификации уровня конкуренции. На первом месте среди арабских стран стоял Египет, с общим количеством публикаций равным 464 (19,27%) документам, в то время как Кувейтский Университет стал самым производительным институтом, издав 158 (6,56%) документов. Средний уровень цитирования на один документ, изданный в арабских странах, составил 13,25, а *h*-индекс - 64. В соответствии с выбранными документами, туберкулез (230; 9,55%), малярия (223; 9,26%), и гепатит (189; 7,8%) были тремя самыми изучаемыми инфекционными заболеваниями.

Заключение: Представленные данные свидетельствуют о том, что некоторые арабские страны вносят значительный вклад в область изучения инфекционных заболеваний. Однако арабским странам предстоит еще много работать, чтобы ликвидировать пробел в этой области. По сравнению с неарабскими странами Ближнего Востока, количество изданных в арабских странах исследований было значительным, но для повышения качества этих исследований необходимо предпринимать больше усилий. Следует стимулировать и правильно направлять будущие исследования в этой области.

Translated from English version into Russian by tatiana_com, through

Evaluación de la productividad en investigación de los países árabes en el campo de las enfermedades infecciosas utilizando la base de datos de la Web of Science

Waleed M. Sweileh, Samah W. Al-Jabi, Alaeddin Abuzanat, Ansam F. Sawalha, Adham S. AbuTaha, Mustafa A. Ghanim, Sa'ed H. Zyoud

Resumen

Antecedentes: Es necesaria una mejor comprensión de los estudios publicados en el campo de las enfermedades infecciosas con el fin de abordar los problemas que puedan plantear en el futuro y de limitar la propagación de microorganismos multirresistentes. El objetivo del presente estudio consistía en el análisis cualitativo y cuantitativo de la investigación realizada en el campo de las enfermedades infecciosas en los países árabes y su comparación con la llevada a cabo en países no árabes.

Métodos: Los documentos publicados en los países árabes dentro de la categoría de investigación "enfermedades infecciosas" se han extraído y analizado utilizando la base de datos de la Web of Science.

Resultados: El número total de documentos publicados en el campo de las enfermedades infecciosas hasta el año 2012 ha sido de 227.188en todo el mundo. En los países árabes se ha publicado un total de 2.408 documentos en el campo de las enfermedades infecciosas, lo que representa el 1,06 % de la producción mundial en investigación. La producción en investigación de los países árabes en el campo de las enfermedades infecciosas ha sido baja durante décadas. No obstante, durante los últimos diez años se ha observado un aumento por cinco. Los países árabes ocupaban los puestos del 56 al 218 de la Standard Competition Ranking (SCR) de publicaciones en el campo de las enfermedades infecciosas a nivel mundial. Egipto, con una publicación total de 464 (19,27%) de documentosocupaba la primera posición de entre los países árabes, mientras que la Universidad de Kuwait fue la entidad más productiva con un total de 158 (6,56%) documentos. La media de referencias bibliográficas por documento publicado en los países árabes fue de 13,25 y el índice *h* de 64. Las principales enfermedades infecciosas estudiadas fueron la tuberculosis (230; 9,55%), la malaria (223; 9,26%) y la hepatitis (189; 7,8%), según los documentos recuperados.

Conclusión: Los presentes datos revelan que algunos países árabes contribuyen de manera significativa al campo de las enfermedades infecciosas. No obstante, deben trabajar más para salvar la brecha en este terreno. En comparación con los países no árabes de Oriente Medio, la producción en investigación de los países árabes ha sido elevada, sin embargo se necesitan más esfuerzos para mejorar su calidad. Se debería fomentar y dirigir correctamente investigaciones futuras.

Translated from English version into Spanish by Raquel Bentué, through

TRANSLATORS
WITHOUT BORDERS
